# Assessment of Cracking Development in Concrete Precast Crane Beams Using Optical and Deep Learning Methods

**DOI:** 10.3390/ma18040731

**Published:** 2025-02-07

**Authors:** Marek Słoński

**Affiliations:** Faculty of Civil Engineering, Cracow University of Technology, ul. Warszawska 24, 31-155 Kraków, Poland; marek.slonski@pk.edu.pl; Tel.: +48-12-628-2562

**Keywords:** concrete, precast, crane beam, crack, digital image correlation, convolutional neural network, U-Net, segmentation

## Abstract

The longevity and safety of concrete precast crane beams significantly impact the operational integrity of industrial infrastructure. Assessment of surface cracks development in concrete structural elements during laboratory tests is performed mainly by applying standard tools such as linear-variable-differential transformers and strain gauges. This paper presents a novel assessment methodology combining deep convolutional neural network for image segmentation with digital image correlation method to evaluate the structural health of precast crane beams after more than fifty years of service. The study first outlines the adaptation of the deep learning U-Net architecture for detecting and segmentation of surface cracks in crane beams. Concurrently, DIC technique is employed to measure surface strains and displacements under load. The integration of these technologies enables a non-destructive, accurate, and detailed analysis, facilitating early detection of deterioration that may compromise structural safety. Initial results from field tests validate the effectiveness of our approach, demonstrating its potential as a tool for predictive maintenance of aging industrial infrastructure.

## 1. Introduction

The assessment of concrete precast crane beams using advanced imaging and machine learning techniques represents a significant change from traditional methods in structural health monitoring. This shift is driven by the need to address the inherent limitations of manual inspection methods, which have long been the standard practice for assessing the structural integrity of such critical components.

Manual inspections, while invaluable for the detailed assessment they provide, are fraught with several limitations that compromise their effectiveness and efficiency. Manual inspections are heavily based on the experience and judgment of the inspectors. This subjectivity can lead to inconsistencies in the assessment outcomes, with potential cracks being overlooked or non-critical anomalies being overemphasized. These inspections are labor-intensive and time-consuming, often requiring scaffolding, facility closure, and extensive human resources, which can lead to significant operational disruptions. The need for physical access to inspect structural elements, especially in large or complex installations such as crane beams, poses safety risks to inspection personnel, particularly in environments with high or difficult-to-reach structures. Manual methods do not scale well with the increasing number and complexity of infrastructures. They become impractical as the frequency of inspections increases or as more detailed and frequent data collection is required for predictive maintenance. The time taken to schedule, conduct, and analyze manual inspections can lead to delays in identifying and addressing structural problems, potentially exacerbating problems that could have been mitigated if detected earlier.

Given these significant drawbacks, there is a pressing need for more reliable, efficient and objective methods to monitor structural health. This research addresses these challenges by leveraging digital image correlation (DIC) and deep learning models, specifically U-Net variants, to provide a non-destructive, automated, and precise method for detecting and analyzing cracks in concrete beams. Incorporating these technologies improves both the accuracy and effectiveness of damage identification while significantly minimizing operational interruptions and safety hazards linked to conventional manual inspections.

Assessment of concrete precast crane beams using optical techniques such as digital image correlation (DIC) and deep learning methods such as a convolutional neural network (CNN) involves leveraging advanced image processing and machine learning methods to assess structural integrity. This approach is part of a broader trend in structural health monitoring, where non-contact methods are increasingly used to assess and monitor infrastructure. The integration of DIC and CNN can enhance the precision and efficiency of detecting and quantifying damage to crane beams, which are critical components in construction and industrial settings.

Concrete structural elements, such as beams, columns and slabs located in industrial building facilities, have been experiencing rapid deterioration due to load and environmental impact [1]. For example, a large proportion of precast post-tensioned structures [2], especially those made of segments in several European countries, have been in service for more than 50 years [3,4,5]. As these structures age or are exposed to various environmental factors, cracks often develop in them, which if not addressed promptly, can compromise the structural integrity and safety of the entire structure.

Traditional methods of evaluating and characterizing these cracks often involve manual inspections, which can be time-consuming, labor intensive, and prone to human error. Furthermore, the subjective nature of manual inspections can lead to inconsistencies in crack assessment, potentially leading to overlooked threats or unnecessary repairs [6,7,8].

The detection and characterization of surface defects, such as cracks in concrete, plays a crucial role in vision-based structural health monitoring systems and in the evaluation of the condition of civil infrastructure. These defects can serve as indicators of ongoing severe degradation processes within a structure. To address this, several strategies have been proposed that use computer vision and image processing methods for the automatic detection of surface defects [9,10,11].

In recent years, rapid advances in computer vision and deep learning architectures for semantic segmentation [12] have opened new avenues for automating and improving the concrete crack detection and evaluation process. Among the various deep learning architectures, the U-Net model has emerged as a promising tool for image segmentation tasks, which makes it particularly suitable for delineating and quantifying cracks in concrete surfaces. By automating the process, we can achieve faster, more consistent, and potentially more accurate assessments of concrete cracks. This approach not only prolongs the lifespan and ensures the safety of structures, but it also results in reduced costs for maintenance and repairs.

Furthermore, as urban areas continue to expand and the number of concrete structures increases, the need for efficient and reliable crack detection systems becomes even more pressing. Using the capabilities of the U-Net model, we can address this growing demand and pave the way for smarter and safer urban development.

To bridge the gap between conventional methods and modern needs, this paper introduces a hybrid approach that combines advanced imaging techniques and machine learning models. This approach aims to improve the precision, efficiency and safety of structural assessments.

This paper is organized as follows. In Section 2, the related works are reviewed and presented. Section 3 presents the novel methodology developed in this work. In Section 4, a comparison of U-Net-based models for the semantic segmentation of cracks is given. Section 5 presents the application of the methodology for the assessment of the concrete precast crane beams. Finally, in Section 6, the most relevant conclusions are drawn.

## 2. Related Works

The evolution of techniques for the detection and characterization of concrete cracks has been marked by significant advancements in computer vision and image processing technologies. This section reviews various methods, highlighting the transition from traditional manual approaches to sophisticated automated systems that employ deep learning for enhanced accuracy and efficiency. To facilitate a clearer understanding of the diverse methodologies and their respective contributions to the field, a tabular summary is provided below.

Standard image-based approaches to the detection and characterization of concrete cracks on the surface of structural elements are based on computer vision methods and image processing techniques integrated into custom-designed methods tailored to specific types of images. These approaches have been extensively presented and evaluated in numerous recent survey articles, for example in [10,11,13,14]. Despite saving time, these approaches often require manual recalibration for different datasets due to their algorithmic rules, which limit their generalizability. This recalibration process could be both laborious and complex, as shown in [15].

On the other hand, a variety of alternative techniques rooted in deep learning have been proposed. These methods can autonomously learn rules from datasets, addressing a key limitation of traditional analysis techniques. Furthermore, adapting an algorithm to incorporate new data generally entails merely augmenting the training set with new samples, while most other training and model parameters remain unchanged. Numerous deep learning approaches have been introduced to segment concrete cracks, employing various methods such as patch-based techniques and semantic segmentation techniques, among others. Islam et Kim in [16] propose an autonomous crack identification method based on a convolutional neural network (CNN) model. It comprises a fully convolutional neural network for crack segmentation, and VGGNet is utilized as the backbone. The results show that the method developed is very efficient for the classification of concrete cracks, attaining a performance of approximately 92% for the recall. Mousavi et al. in [17] used an optimized U-shaped convolutional neural network with a novel training strategy for the segmentation of concrete cracks. The proposed model is an encoder-decoder model that uses EfficientNet-B7 as the encoder and the modified expansion path of U-Net as the decoder.

Recently, ED-CNNs have been developed for semantic image segmentation [18]. Motivated by these accomplishments, numerous recent investigations have devised ED-CNN-based models aimed at automatic semantic segmentation of concrete cracks [19,20]. Dung and Anh [21] proposed a segmentation method using a VGG16-based encoder. Yang et al. [22] applied the fully convolutional network (FCN) to enhance the effectiveness of crack detection at the image level and the cell level of the grid. The findings indicate that the use of FCN is both practical and adequate for detecting and assessing cracks. In the paper by Pu et al. [23] research on recognizing crack patterns in concrete through images is presented using a convolutional neural network and an encoder–decoder based component for semantic segmentation. Shen et al. in their paper [24] propose an enhanced method based on the open source Deeplabv3+ model and name it Deeplabv3+ BDF in line with the optimization strategy employed. Deeplabv3+ BDF initially replaces the original Xception backbone with MobileNetv2 and then substitutes replace all traditional convolutional layers with depthwise separable convolutions to create a lightweight model. Razveeva et al. in [25] present an examination of the geometric properties of cracks and delamination in aerated concrete products utilizing convolutional neural networks. Guo et al. in [26] describe the monitoring and automatic characterization of cracks in a hardening-resistant cementitious compound (SHCC) by intelligently analyzing photographs. Xu et al. in [27] propose a data set benchmark for the segmentation of concrete cracks at the pixel level, using a deep learning model. The suggested benchmark for the concrete crack dataset offers engineering importance and can enhance model efficiency by carefully choosing suitable techniques, such as expanding the dataset size or refining the model design, while also reducing the time needed for dataset annotation. Forest et al. in [28] propose applying the explainable artificial intelligence (XAI) methodology to segment and monitor surface cracks. Ahmadi et al. in [29] evaluated the effectiveness of two deep learning models, SAM and U-Net, to identify cracks in concrete structures. The findings suggest that each model has distinct strengths and weaknesses in detecting various types of cracks.

König et al. in [30] reviewed recent studies in this research area. In particular, semantic segmentation techniques have exhibited state-of-the-art (SOTA) performance in crack segmentation tasks on concrete surface images [31,32,33]. Zhou et al. in [34] review recent developments in deep learning-based crack segmentation methods and investigate their performance under the impact of different image types. Deng et al. in [35] conducted a literature review, focusing mainly on qualitative damage evaluation or damage segmentation.

Most approaches for semantic segmentation of concrete cracks utilize the encoder-decoder deep neural network structure inspired by the superior accuracy of the U-Net architecture for medical applications [36]. This architecture improves the original fully convolutional network (FCN) methodology [37]. Other popular encoder-decoder architectures like Segnet [38] and DeepLab [39] were also introduced.

In U-Net, skip connections facilitate gradient flow and enable information exchange between the down-sampling and up-sampling paths by linking the corresponding encoder and decoder layers. U-Net processes an input image and assigns each pixel to one of several classes, thus segmenting the image into distinct regions. For surface concrete crack segmentation, these classes differentiate between crack and non-crack areas. The result is a pixel-wise map from the U-Net, which provides precise segmentation of the target regions.

Various adaptations have been created to improve the effectiveness of the initial U-Net design. Dense U-Net links the outputs of all convolutional blocks to the inputs of the following blocks within each level, promoting feature reuse and mitigating vanishing gradients. The Inception U-Net uses multiple convolutional kernel sizes in parallel paths at each level, providing robustness to images of varying scales.

Attention U-Net integrates an attention module within each skip connection, allowing the network to focus more on important spatial regions in an image and less on less relevant ones. Rao et al. in [40] propose a novel deep learning framework to detect cracks and then estimate crack widths in images of concrete surfaces. The proposed framework uses Attention Recurrent Residual U-Net (Attention R2U-Net) with Random Forest regressor to predict the crack width. Hang et al. in [41] present an attention-based feature fusion network (AFFNet), with a backbone residual network (ResNet101) enhanced with two modules of the attention mechanism for automatic detection of concrete cracks at the pixel level. Evaluations against other leading models demonstrate the proposed method high efficiency and precision.

Lastly, residual U-Net incorporates residual learning by adding shortcut connections in each layer, which improves gradient flow and operates under the principle that learning a residual mapping is more straightforward than learning the original one.

Shamsabadi et al. in [42] introduced a vision transformer (ViT)-oriented framework to detect cracks on asphalt and concrete surfaces. In contrast to CNN-based models such as DeepLabv3+ and U-Net, our TransUNet model, which incorporates a CNN-ViT architecture, demonstrated enhancements in mean Intersection over Union (IoU) by approximately 61% and 3.8% on the original images of the corresponding datasets. This improvement was observed particularly among images exhibiting small and multi-scale crack features.

In a study [43], Wang and Su introduce an innovative SegCrack model designed for pixel-wise crack segmentation, utilizing a Transformer encoder with a hierarchical structure to produce multiscale features. This model employs a top-down pathway enriched with lateral connections to incrementally up-sample and integrate features from the encoder’s deepest layer.

Past research on concrete crack detection has introduced several adaptations of the U-net architecture using Transformer architecture to enhance performance. Xiang et al. in [44] proposed a dual encoder network that combines transformers and convolutional neural networks (DTrC-Net). DTrC-Net architecture was designed to effectively capture both the local features and the global context of the crack images. To improve the merging of features between adjacent layers and the coding-decoding stages, the network incorporates a feature fusion module alongside a residual path module. In general, DTrC-Net showed superior performance in crack segmentation accuracy and exhibited exceptional generalization capabilities in intricate environments compared to other state-of-the-art networks.

However, the specific impact of these modifications on the segmentation accuracy of concrete crack images remains uncertain due to the lack of an impartial comparison. Shi et al. in [45] compared semantic segmentation networks for crack detection in construction materials. Li et al. in [46] made a survey of deep learning-based pixel-level crack image segmentation methods. They grouped the methods into 10 topics based on the backbone network architecture. The comparison of the application of convolutional neural networks for concrete crack detection is presented in [47].

This study addresses this gap by evaluating three prominent U-net variants in the SDNET2018 dataset, aiming to discern their influence on segmentation performance and balancing these findings against training, evaluation time, and computational complexity.

To facilitate a clearer understanding of the diverse methodologies and their respective contributions to the field, a tabular summary is provided in Table 1.

## 3. Materials and Methods

### 3.1. Overview

**Figure 1:**
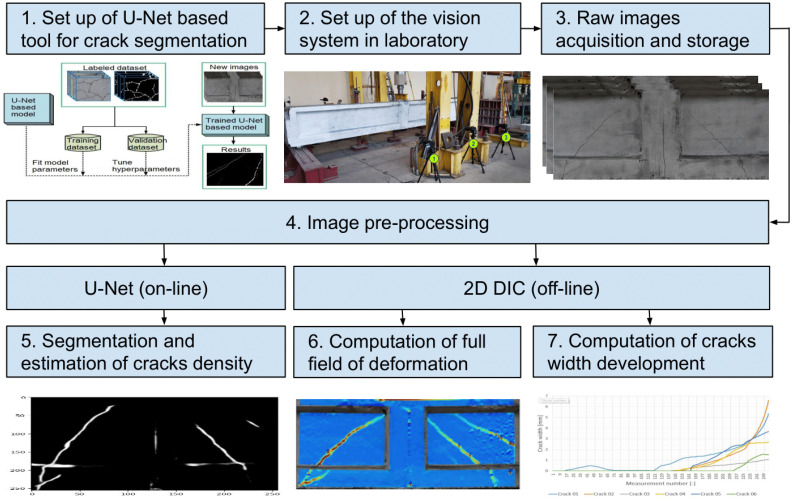
A diagram illustrating the experimental–computational framework employed in this study.

The methodology depicted in Figure 1 involves steps that facilitate a comprehensive evaluation of cracking in the beams through advanced image processing techniques. Here is a detailed description of each step illustrated in the diagram.

1. Setup of a U-Net based model for crack segmentation.
  This step involves off-line training of selected U-Net based deep learning models for semantic segmentation of surface cracks using available datasets from the Internet. The trained best U-Net based model is set up to identify and segment cracks in images of crane beams on-line during the laboratory experiments.
2. Setup of the vision system in laboratory.
  The vision system, which includes high-resolution DSLR cameras and appropriate lighting setups, is installed in a laboratory environment. This setup is crucial to capture detailed images of the crane beams under examination. The system is calibrated to ensure that the images obtained are suitable for both U-Net segmentation and DIC analysis.
3. Raw images acquisition and storage.
  High-quality raw images of the crane beams are captured using the laboratory vision system for subsequent processing. The image acquisition is done under controlled lighting to minimize noise and ensure that the finest cracks can be detected.
4. Image pre-processing.
  The raw images (optionally) undergo pre-processing, which includes noise reduction, contrast enhancement, and scaling to prepare the images for analysis by both the U-Net model and the DIC software. Concrete images may contain noise due to the conditions under which the photos are taken. The application of a Gaussian blur filter smooths out the images, reducing pixel-level noise. It may help minimize false positives in crack detection. The contrast enhancement technique was used to improve the visibility of the cracks by sharpening the intensity differences between the cracks and the surrounding concrete. The images were resized to ensure consistent input size for the models.
5. Segmentation and estimation of cracks density.
  The preprocessed images are fed into the U-Net model, which performs segmentation of the cracks online. Following segmentation, the density of the cracks is calculated, providing quantitative data on the extent of cracking, which is critical for assessing the structural capacity of the beams.
6. Computation of full field of deformation.
  Using 2D DIC software, the deformation of the crane beams under load is analyzed off-line. This software computes the displacement fields across the beam’s surface from the images, allowing for a detailed understanding of how the beams deform under loading.
7. Computation of cracks width development
  The final step involves using the DIC results to calculate the development of crack widths over time or under varying load conditions. This analysis helps to understand the behavior of cracks during the loading process and contributes to predicting the future behavior of these structural elements.

This method combines U-Net with DIC to improve the identification and segmentation of structural flaws and to deliver an in-depth evaluation of the mechanical performance of the beams, thereby presenting a strong strategy for monitoring structural health in civil engineering.

### 3.2. Digital Image Correlation

Digital Image Correlation (DIC) is a very useful method for evaluating structural element deformations [48,49,50]. DIC method was first introduced at University of South Carolina at the beginning of the 1980s as described in [51,52]. DIC method works by examining successive images recorded in the process of the deformation of an element under loading and establishing a correspondence between the pixel coordinates of the undeformed image and the pixel coordinates of the deformed image through correlation analysis, which gives the maximum correlation between the reference image and the deformed image. This mapping is utilized for determining full-field strains [53].

**Figure 2:**
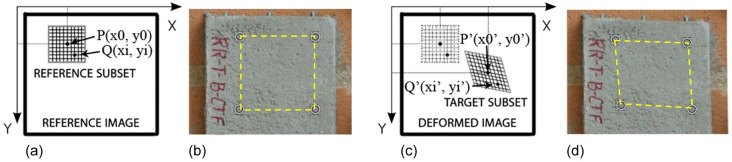
Illustration of the basic principle of the digital image correlation method: Sketch of the subset (**a**,**c**) and measurement points on the surface of a specimen (**b**,**d**) before (**a**,**b**) and after (**c**,**d**) deformation [54].

The nodes of the grid based on the defined image regions are paired and recognized as that corresponding to the maximum value of the correlation coefficient. This correlation measure is computed between the reference subset “f” and the target subset “g”, with identical dimensions and are M × N pixels employing the zero-mean normalized cross-correlation formula specified in Equation (1). The basic principle of digital image correlation is illustrated in Figure 2.

(1) $${\mathrm{CC}}^{\mathrm{ZMN}}=\frac{\sum_{i=1}^{M}\sum_{j=1}^{N}\left[\left(f\left(i,j\right)-u_{f}\right)\times (g\left(i,j\right)-u_{g}\right)]}{\sqrt{\sum_{i=1}^{M}\sum_{j=1}^{N}{\left(f\left(i,j\right)-u_{f}\right)}^{2}\times \sum_{i=1}^{M}\sum_{j=1}^{N}{\left(g\left(i,j\right)-u_{g}\right)}^{2}}},$$

where $u_{f}$ is the intensity of the reference subset and $u_{g}$ is the intensity of the target subset form.

### 3.3. Deep Convolutional Encoder-Decoder Architectures

A deep convolutional encoder-decoder architecture is a type of neural network that is commonly used for computer vision tasks [55]. It consists of two main components: An encoder and a decoder. The encoder component of a deep convolutional encoder-decoder network is responsible for extracting meaningful features from the input data. It typically consists of multiple convolutional layers followed by pooling layers to reduce the spatial dimensions of the feature maps. The encoder gradually learns to encode the input data in a lower-dimensional representation, capturing important information in the process.

The decoder component of the network takes the encoded representation and reconstructs the output data. It usually consists of upsampling layers to increase the spatial dimensions of the feature maps, followed by convolutional layers to refine the reconstructed output. The decoder gradually learns to decode the encoded representation back into the original input data, generating the desired output. By combining the encoder and decoder components, a deep convolutional encoder-decoder network can effectively perform tasks such as image segmentation, image-to-image translation, and image generation, by learning to encode and decode the relevant information in the input data.

In this paper, 3 architectures are applied: U-Net, Attention U-Net and TransUNet. They are briefly described in the following subsections. The number of parameters and the number of convolutional layers are presented in Table 2.

#### 3.3.1. U-Net Architecture

U-Net is a convolutional neural network architecture for fast and precise image segmentation. It was proposed by Olaf Ronneberger, Philipp Fischer, and Thomas Brox in 2015 [36]. The network is based on the fully convolutional network (FCN) which was introduced by Long et al. in 2014 in [37]. Its architecture was modified and extended to work with fewer training images and yield more precise segmentations. U-Net is widely used in the field of medical image segmentation, where it has achieved state-of-the-art performance.

**Figure 3:**
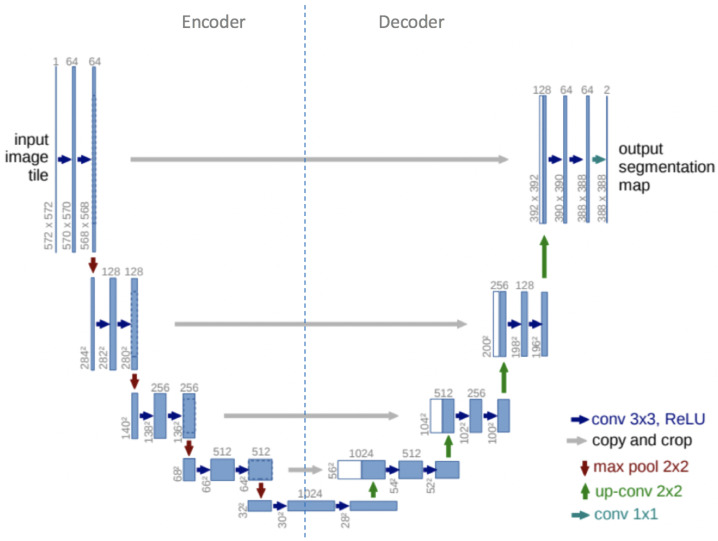
U-Net architecture, taken from the original paper [36].

U-Net architecture is comprised of a contracting path to capture local details and an expanding path for global context. The contracting path includes convolutional and pooling layers, while the expanding path features upsampling and convolutional layers. Furthermore, U-Net uses skip connections to maintain spatial detail and enhance segmentation precision. The U-Net architecture is presented in Figure 3.

#### 3.3.2. Attention U-Net

**Figure 4:**
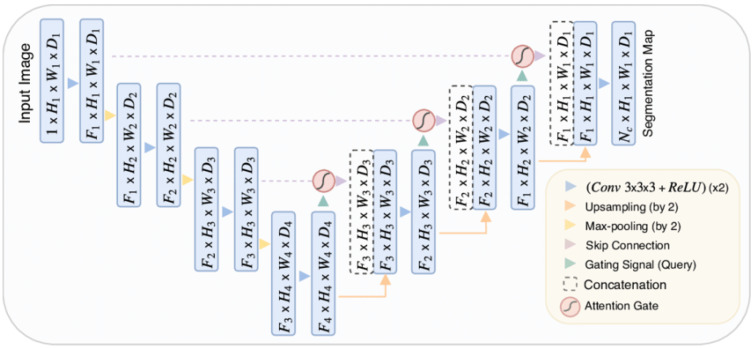
Attention U-Net architecture, taken from the original paper [57].

**Figure 5:**
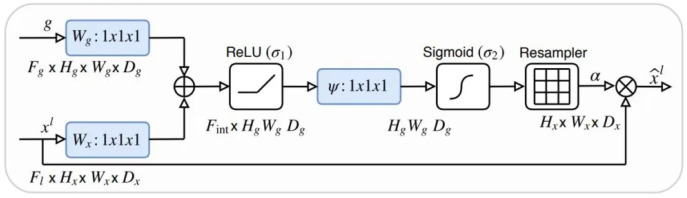
The block diagram of attention gate in Attention U-Net architecture, taken from the original paper [57].

The Attention U-Net architecture is an extension of the traditional U-Net architecture that incorporates the concept of attention mechanisms introduced by Bahdanau et al. in 2014 in [56]. The Attention U-Net architecture was proposed by Ozan Oktay et al. in 2018 in [57]. It is a variant of the original U-Net architecture that incorporates attention mechanisms. The architecture is presented in Figure 4 and the attention gate diagram is shown in Figure 5.

It was designed to improve the performance of U-Net in tasks where capturing long-range dependencies and focusing on relevant image regions is crucial, such as image segmentation and object detection. Attention U-Net was introduced to the research community to enhance the model’s ability to handle fine-grained details and complex patterns.

The inclusion of an attention mechanism in Attention U-Net enhances the ability of the model to concentrate on essential areas of the input image, thereby increasing its segmentation precision. This approach is modeled on the human visual system, which prioritizes certain parts of an image over others. Integrating attention mechanisms within the U-Net framework has led to notable improvements in model performance in a range of medical image segmentation applications, including tumor and organ segmentation.

#### 3.3.3. TransUNet

**Figure 6:**
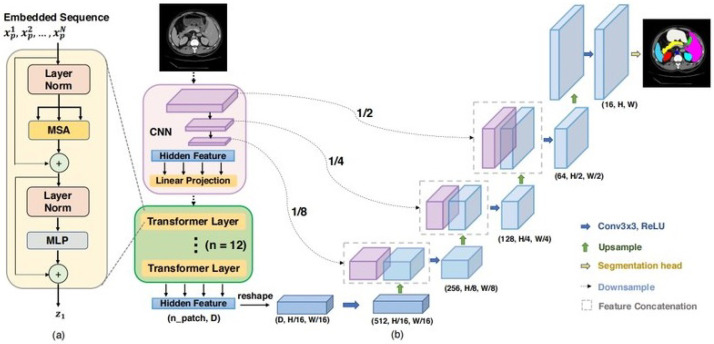
TransUNet architecture: Transformer layer framework (**a**), TransUNet layer structure (**b**), taken from the original paper [59].

The TransUNet architecture combines the strengths of transformer-based models introduced by Vaswani et al. in 2017 in [58] (for context and feature extraction) with the architecture of a U-Net (for spatial segmentation tasks). This architecture was proposed by Chen et al. in 2021 in [59]. It can be used for tasks such as semantic segmentation, object detection, or medical image analysis, where capturing fine-grained spatial details and global context is essential. The architecture is presented in Figure 6.

The choice of U-Net variants - specifically the standard U-Net, Attention U-Net, and TransUNet - for this study was guided by their distinct architectural strengths, which are particularly beneficial for the complex task of segmenting concrete cracks. Each of these models offers unique advantages in processing images characterized by subtle and intricate crack patterns, which are common in aged concrete structures.

The standard U-Net model is renowned for its efficiency and effectiveness in image segmentation tasks, particularly when dealing with limited data. Its architecture leverages a symmetric encoder-decoder structure with skip connections that help preserve spatial hierarchies between image features at different scales. This capability is crucial for detecting and delineating fine cracks, ensuring that even minor discrepancies on the surface of the concrete are captured and analyzed.

Attention U-Net extends the standard U-Net by incorporating attention mechanisms, which are particularly adept at refining the focus on relevant features while suppressing less useful information. This model improves the precision of segmentation in images where cracks are surrounded by complex textures or noise, a common scenario in deteriorating infrastructure. By directing the model’s focus towards salient features, Attention U-Net is better equipped to isolate and characterize cracks with higher accuracy, making it ideal for detailed structural assessments where every millimeter counts.

TransUNet integrates the robust feature extraction capabilities of Transformers with the spatial context preservation of U-Nets. This hybrid model excels at capturing both the global and local context, a necessary feature for comprehensive crack analysis across varying scales and intensities. Transformer components address the challenge of long-range dependencies, allowing for more consistent segmentation in larger crack zones and improving the generalizability of the model to different types of concrete surfaces and damage patterns.

The models were chosen for their technical strengths, supported by initial tests that showed that they outperformed other deep learning frameworks in crack segmentation tasks. For example, TransUNet demonstrated exceptional capability in maintaining high recall rates, crucial for ensuring that no potential crack is missed, which is paramount in predictive maintenance and safety assessments. Attention U-Net showed higher precision, which is critical in minimizing false positives, thus avoiding unnecessary inspections and repairs.

Through these enhanced capabilities, the selected variants of U-Net facilitate a more accurate, reliable, and cost-effective way to monitor and maintain the aging infrastructure, in line with the goals of advanced structural health monitoring systems. Thus, this strategic selection directly contributes to the overarching aim of the investigation: to improve the precision and reliability of concrete crack detection in support of structural integrity assessments.

### 3.4. Dataset

The dataset used for this study consists of high-resolution images of concrete surfaces. These images were manually annotated to provide ground-truth labels for crack regions.

Before feeding the images into the model, several preprocessing steps were applied. These include resizing the images, normalization, and data augmentation techniques to improve the model’s robustness.

**Figure 7:**
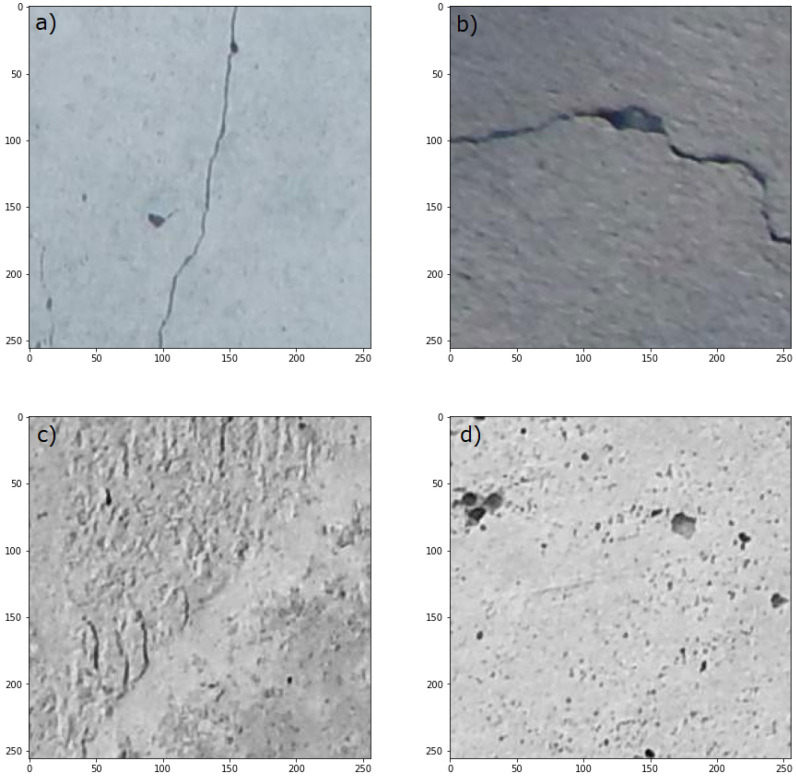
Images of cracked (**a**,**b**) and uncracked concrete pavements (**c**,**d**), sampled from SDNET2018 dataset [60].

To evaluate the performance of the models, we us ed 800 images of cracked concrete elements randomly selected from the open source data set called SDNET2018 [60]. SDNET2018 contains more than 56,000 images of cracked and non-cracked concrete bridge decks, walls, and pavements. The data set includes cracks as narrow as 0.06 mm and as wide as 25 mm. The data set also includes images with a variety of obstructions, including shadows, surface roughness, scaling, edges, holes, and background debris. Four images of cracked and uncracked concrete pavements from the benchmark dataset are shown in Figure 7.

While SDNET2018 is a widely used benchmark, it has several shortcomings. The dataset predominantly features images captured under controlled conditions, which may not represent the variability encountered in real world scenarios. This limitation could potentially introduce a bias toward specific types of cracks or surface conditions, affecting the generalizability of the model. The images in SDNET2018 do not adequately capture the diverse environmental impacts that real-world structures undergo, such as weathering or chemical degradation. This exclusion can lead to models that perform well in laboratory settings, but less so in harsh or outdoor environments.

The are some challenges in annotating the dataset (inaccuracies and subjectivity) that can influence the accuracy and effectiveness of the resulting models. These challenges were addressed using an automated tool to pre-annotate images based on common crack patterns, which were then manually refined. Cracks were annotated using the Segments-ai application [61].

**Figure 8:**
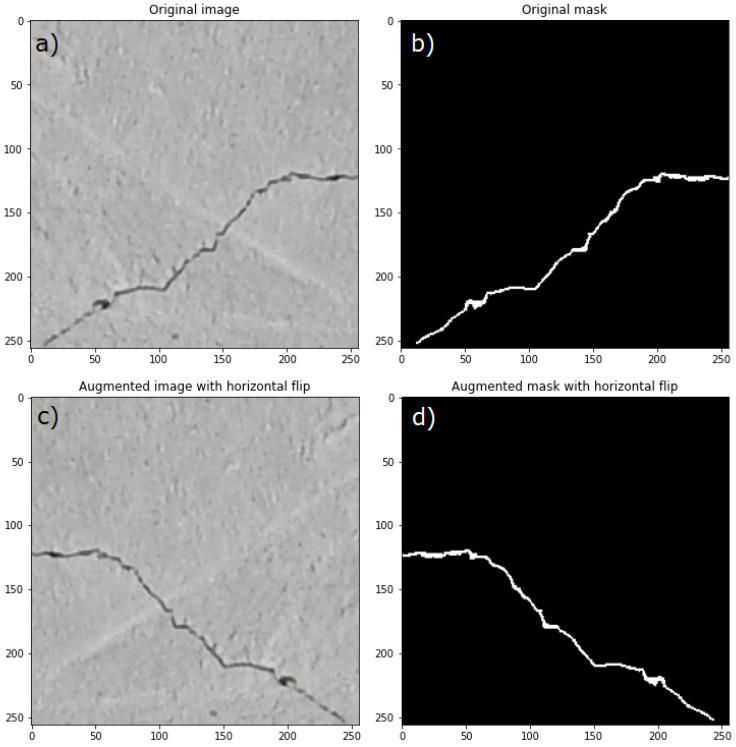
Example of new image and mask (**c**,**d**) obtained from original image and mask (**a**,**b**) by horizontal flip, taken from [62].

**Figure 9:**
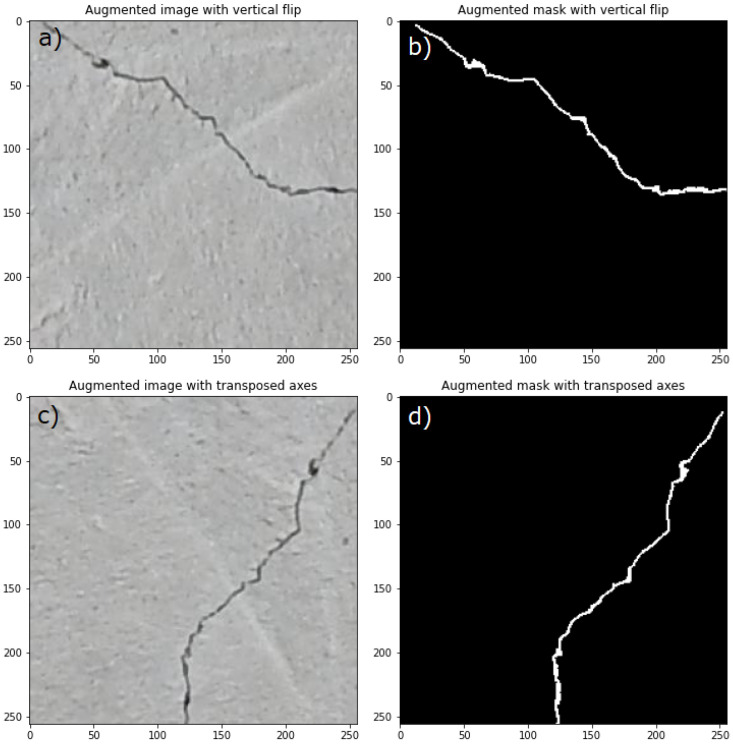
Examples of new images and masks obtained by vertical flip (**a**,**b**) and by transpose (**c**,**d**), taken from [62].

In Figure 8, an example of a new image and a mask obtained from the original image and mask by horizontal flip are shown. Two examples of new images and masks obtained by vertical flip and transpose are presented in Figure 9.

The data set was divided into a training data set (2560 images) and a validation data set (640 images). The created dataset (accessed on 20 January 2024) is available at: https://www.kaggle.com/datasets/jakubniemiec/concrete-crack-images.

Data augmentation (horizontal, vertical, and flipping) was used to increase the number of labeled images to 3200 images.

### 3.5. Evaluation Metrics for Crack Segmentation

The true measure of the efficacy of any model lies in its evaluation against robust and relevant metrics. In this section, we introduce and elucidate the key metrics used to gauge the performance of the U-Net model in the context of concrete crack assessment. The selection of these metrics is based on both their statistical robustness and their applicability to real-world scenarios, guaranteeing that our evaluation is both theoretically robust and practically significant. From assessing the model’s accuracy in detecting minute cracks to its precision in characterizing their parameters, each metric provides a unique lens through which to view the model’s capabilities. By delving into these evaluation metrics, we aim to provide a transparent and comprehensive understanding of the standards against which our research findings have been benchmarked.

**Figure 10:**
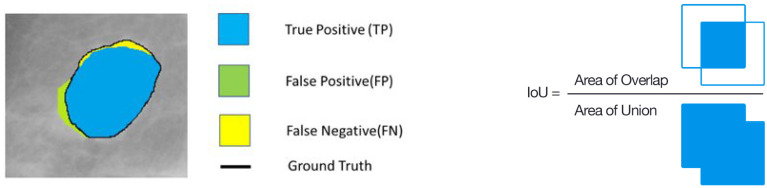
Graphical representation of: confusion matrix (**left**) and Equation (5) (**right**).

The effectiveness of crack detection algorithms in segmentation was evaluated using a confusion matrix, as detailed in Table 3 and represented graphically in Figure 10. This matrix is composed of two rows and two columns that display the pixel-wise classification results on the test dataset, where the rows indicate the actual classes and the columns denote the predicted classes. Specifically, TP signifies crack pixels that are correctly classified as cracks, TN indicates non-crack pixels accurately recognized as non-cracks, FP refers to non-crack pixels incorrectly predicted as cracks, and FN represents crack pixels that the model fails to identify, misclassifying them as non-cracks.

To evaluate the performance of the model, four metrics were used:

- Precision—measures the proportion of true positives out of all predicted positives,
- Recall (or Sensitivity)—measures the proportion of true positives out of all actual positives,
- Dice Score Coefficient (DSC) or F1 score is the harmonic mean of precision and recall, balancing both metrics,
- Mean Intersection over Union (mIoU) is a common metric in semantic segmentation tasks, measuring the overlap between predicted and ground truth labels.

These metrics provide a comprehensive evaluation of the model’s ability to accurately segment concrete cracks.

(2) $$Precision=\frac{TP}{TP+FP},$$

(3) $$Recall=\frac{TP}{TP+FN},$$

(4) $$DSC=F1=\frac{2|A\cap B|}{\left|A|+|B\right|}=\frac{2\times Precision\times Recall}{Precision+Recall}=\frac{2TP}{2TP+FP+FN},$$

(5) $$IoU=\frac{\left|A\cap B\right|}{\left|A\cup B\right|}.$$

where:

A—original mask,

B—predicted mask.

### 3.6. Experimental Settings

The models were trained using the supervised learning approach and the cost function is defined as the Dice loss introduced in [63], using the Dice coefficient; see the Equation (4):

(6) $$Dice\;loss=1-DSC=1-\frac{2|A\cap B|}{\left|A|+|B\right|}.$$

The implementation of the models was based on the Keras-Unet-Collection [64]. The experiments were carried out using Google Colab Pro services [65]. The specification description of the Colab environment applied consists of GPU (NVIDIA Tesla A100 Tensor Core), CPU (Intel^®^ Xeon^®^ E5-2630) and RAM (27.3 GB). This advanced hardware set-up facilitated effective experimentation with different deep learning techniques for crack segmentation. To improve the crack segmentation performance of the model, we applied a series of targeted training methodologies. Initially, we used ImageNet for pretraining to initialize the base model parameters, offering general-purpose features as an initial foundation. During simulation, we adjusted the weights in both the convolutional and up-sampling layers, scaling them to enhance the learning of distinctive crack features.

In Table 4, the total number of parameters, the number of trainable parameters and the training time of the three networks is shown. The TransUNet model has substantially more parameters and a longer time than the other two models.

## 4. Comparison of Three U-Net Based Models for Semantic Segmentation of Concrete Surface Cracks

**Figure 11:**
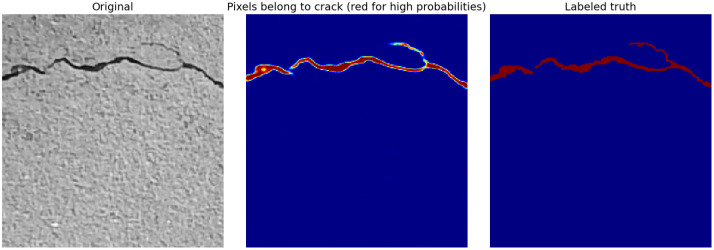
Example of semantic segmentation results for the trained U-Net model.

After training, the models were used to classify each pixel in the crack image of the concrete surface as cracked or non-cracked. For example, Figure 11 presents the segmentation results for the trained U-Net model. The leftmost image shows the original grayscale concrete surface with a visible crack. The middle image presents the prediction results of the U-Net model. It uses a heatmap style where red indicates higher probabilities that pixels belong to a crack, transitioning to blue for lower probabilities. This visualization clearly shows how the model has identified the crack path with varying confidence levels along its length. The rightmost image is the ground truth (labeled data) used to train the model. It marks the actual crack with a solid red color.

The middle image shows that the U-Net model is quite effective in detecting the central, continuous parts of the crack, which are highlighted in bright red. There are areas where the model confidence fades (shown by the yellow to blue gradient), indicating either weaker detection or actual variations in the crack visibility or width.

**Figure 12:**
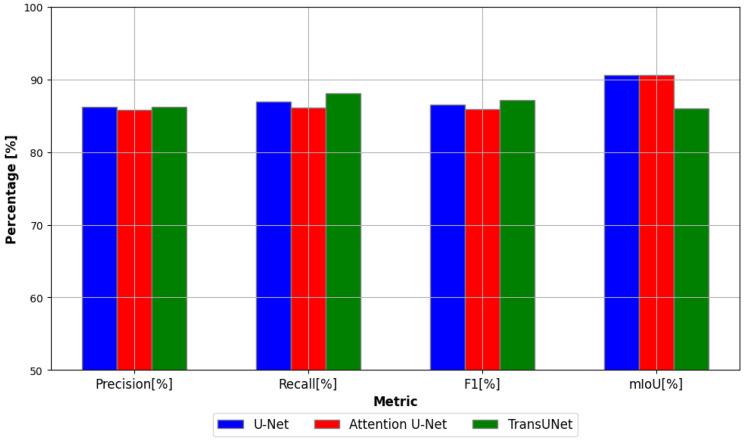
Comparison of model performance metrics.

In Table 5 and in Figure 12, the performance of the three networks is quantitatively compared using four metrics.

In this comparison, Attention U-Net has the highest precision (84.89%), U-Net follows closely with 84.45% and TransUNet has the lowest precision at 83.40%. This indicates that Attention U-Net performs slightly better in reducing false positives compared to the other models, while TransUNet sacrifices some precision, potentially to improve other metrics. In the case of the recall metric, TransUNet stands out with the highest recall (90.92%), outperforming the other models by a significant margin. Attention U-Net and U-Net achieve recall values of 87.71% and 87.16%, respectively.This shows that TransUNet excels at identifying true positives, making it more effective when missing fewer relevant predictions is critical. In this comparison, using the F1 score, TransUNet performs the best with an F1 score of 86.75%, indicating a good balance between precision and recall. Attention U-Net comes second with an F1 score of 85.82%, while U-Net has a slightly lower F1 score of 85.33%. This suggests that while the precision for TransUNet is slightly lower, its high recall helps to maintain a strong overall F1 score.

**Figure 13:**
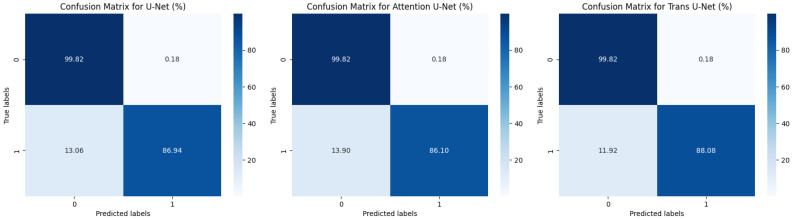
Comparison of confusion matrices.

In Figure 13, the performance of the three networks is quantitatively compared using confusion matrices.

In the case of the Mean Intersection Over Union (mIoU) metric, TransUNet achieves the highest mIoU (88.21%), indicating superior performance in terms of overall accuracy and overlap with ground truth segmentation. Attention U-Net (87.59%) and U-Net (87.35%) have similar performances, trailing just behind TransUNet.

**Figure 14:**
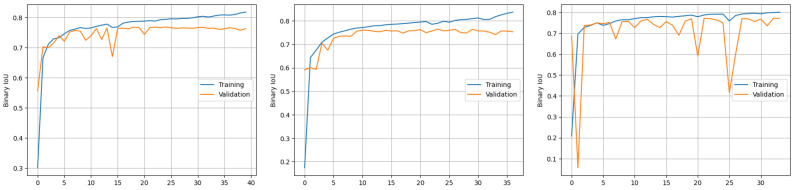
Comparison of binary IoU metrics.

In Figure 14, the performance of the three networks is quantitatively compared using binary IoU metrics.

In conclusion, TransUNet consistently performs the best in most metrics, especially in terms of recall, F1 score, and mIoU. This indicates that TransUNet is a well-balanced model, particularly strong in identifying true positives and providing accurate segmentations. Attention U-Net outperforms U-Net in every metric, particularly in precision, making it a solid improvement over the basic U-Net architecture. U-Net, while the original model, still performs competitively, but is slightly outclassed by its attention-based variant and TransUNet. These insights from Table 5 and the bar graph in Figure 12 indicate that TransUNet is probably the most suitable model for this specific semantic segmentation task, especially when recall and mIoU are prioritized.

**Figure 15:**
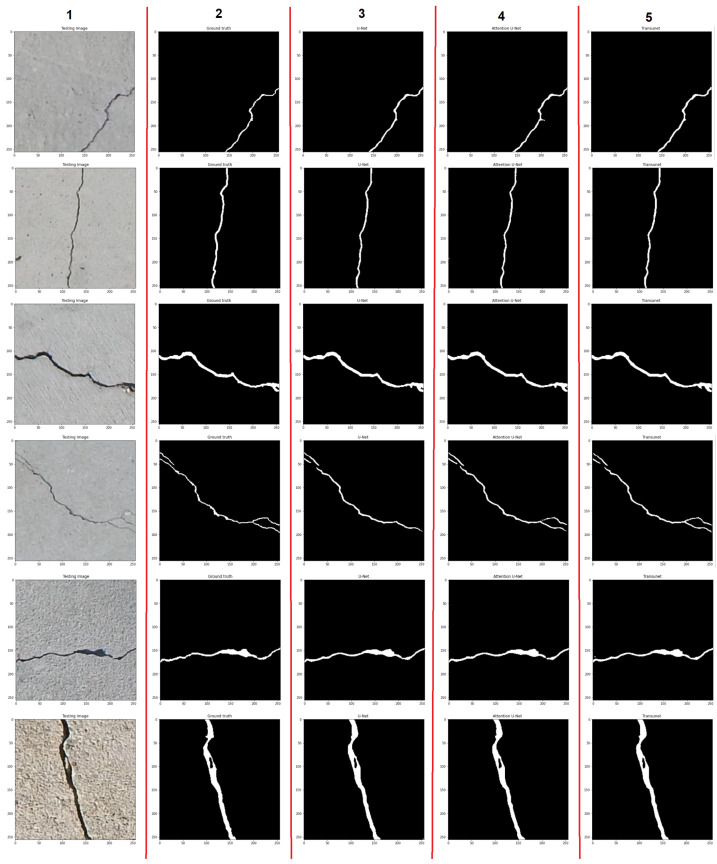
Examples of concrete cracks segmented by 3 models. Column 1: input images with cracks, column 2: annotated ground truth masks, column 3: predicted crack masks by U-Net, column 4: predicted crack masks by Attention U-Net, column 5: predicted crack masks by TransUNet, taken from [62].

In Figure 15 a comparison of the semantic segmentation of the cracks by the three models (U-Net, Attention U-Net, and TransUNet) is presented. In the first column, the selected images that contain cracks are presented. In the second column, the annotated ground-truth masks are displayed. The segmentation results for three networks are shown in columns 3–5, respectively. The segmentation results presented show that trained models are capable of predicting crack pixels with satisfactory accuracy, in a wide width range, from very thin cracks to very thick cracks. In the set of images presented, the continuousness of the cracks is also well maintained. Despite satisfactory predictions, there are few images in which the models fail to correctly classify some pixels as cracks. It is especially visible for the fourth image and the U-Net, where there are some pixels segmented as non-cracked pixels.

**Figure 16:**
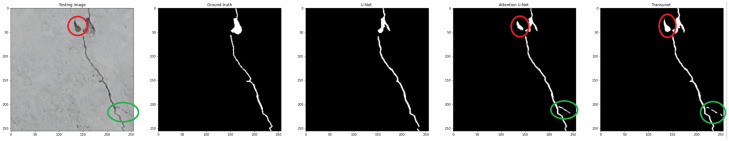
Another comparison of segmentation of concrete crack by 3 models. (**image 1**): input image with crack, (**image 2**): annotated ground truth mask (the red and green marked elements do not appear in the input mask), (**image 3**): predicted crack mask by U-Net, (**image 4**): predicted crack mask by Attention U-Net, (**image 5**): predicted crack mask by TransUNet, taken from [62].

There are also cases where the model performs better than the original mask. In Figure 16, it can be seen that the red and green marked elements do not appear in the input mask (the second image on the left); however, the result of the Attention U-Net and TransUNet model classified these areas as cracked. During the evaluation of the model, these areas were considered incorrectly predicted, but by manually analyzing the images, it can be determined that these areas are correct and can be considered cracked.

**Figure 17:**
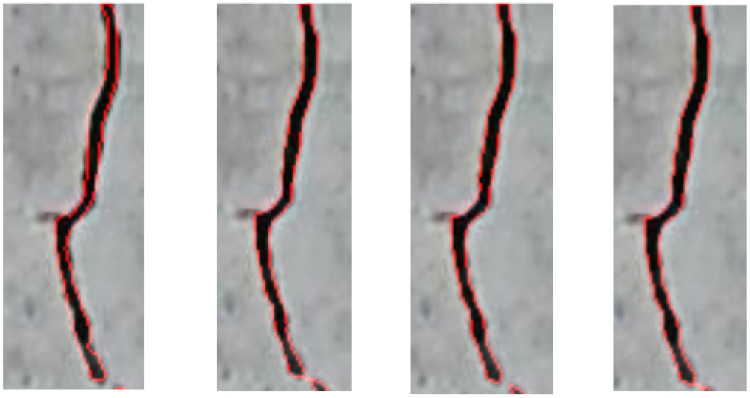
Ground truth mask contour (left) and contours predicted by each model (U-Net, Attention U-Net and TransUNet), taken from [62].

**Figure 18:**
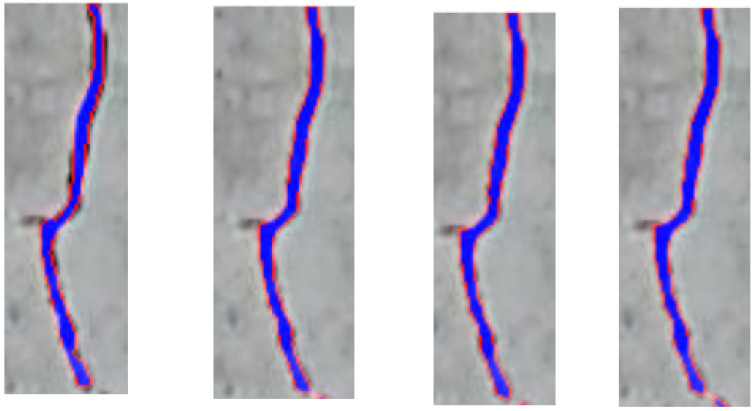
Ground truth mask filling (left) and fillings predicted by each model (U-Net, Attention U-Net and TransUNet), taken from [62].

Figure 17 shows the contours predicted by each model that are more precise than the ground truth mask. Figure 18 shows the fillings predicted by each model. When analyzing the contours and fillings, it is possible to see the inaccuracy of the masks. This is possible because the masks come from the manual labeling of the crack images.

**Figure 19:**
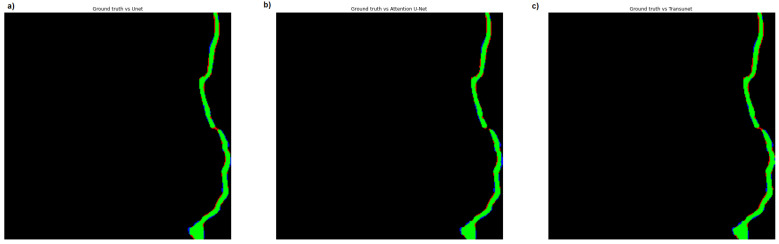
Classification of pixels for each model in comparison to the ground-truth mask: U-Net (**a**), Attention U-Net (**b**) and TransUNet (**c**), taken from [62].

Figure 19 shows the classification of pixels for each model in comparison to the ground-truth mask. The green color represents the common pixels for the ground truth mask and the segmentation results for each model. The red color illustrates pixels predicted by the models as cracked that are not in the ground-truth mask. The blue color represents pixels present in the ground truth mask that are absent in the predicted mask.

TransUNet outperforms others models by combining the advantages of the Transformer architecture with the proven effectiveness of the U-Net framework. The Transformer component of TransUNet allows the model to capture global contextual information, which is crucial for understanding the broader area in which cracks are situated. TransUNet’s ability to integrate features at different scales using both Transformer and convolutional layers results in richer feature representations. The combination of global and local processing in TransUNet makes it robust against variations in image quality, lighting conditions, and crack characteristics, increasing its utility in practical applications where such variability is common.

While TransUNet shows promising results, there is room for improvement, especially in terms of computational efficiency and real-time processing capabilities. Future work could focus on optimizing the model’s architecture to reduce resource consumption without compromising performance. Furthermore, testing the model in a wider range of real-world scenarios, including outdoor environments with varying weather conditions, will be crucial to further validate its effectiveness and readiness for deployment in structural health monitoring applications.

While TransUNet demonstrates superior performance in crack segmentation, its deployment in real-world scenarios requires a critical assessment of its computational demands and scalability, especially in relation to larger datasets and real-time applications. The model’s reliance on deep Transformer layers requires substantial memory and processing power, which can be a limiting factor in environments lacking specialized hardware. Due to its extensive network architecture, training TransUNet on large datasets involves longer durations, potentially making the model less feasible for projects with tight development cycles. As datasets grow in size and complexity, the computational resources required to train TransUNet can become prohibitively expensive. This is particularly challenging for organizations without access to high-performance computing facilities. For real-time crack detection and monitoring applications, the high inference time of TransUNet could be a bottleneck. Real-time processing demands quick turnaround times, which may not be achievable with the current model architecture without significant optimizations.

To address these challenges and improve the practical utility of TransUNet, developing simplified versions of TransUNet, which maintain a balance between performance and computational efficiency, could help reduce the model resource requirements. Leveraging more efficient computational hardware such as FPGAs (Field-Programmable Gate Arrays) or specialized AI accelerators can improve the model’s performance in real-time scenarios. Implementing a hybrid approach that uses lightweight models for initial crack detection in real-time and reserves TransUNet for detailed analysis in batch processing scenarios can optimize resource usage while maintaining high accuracy levels. Future research should focus on addressing these computational challenges to improve the adaptability and efficiency of TransUNet.

## 5. Assessment of Precast Crane Beams Using DIC Method and U-Net Based Model

### 5.1. Overview

**Figure 20:**
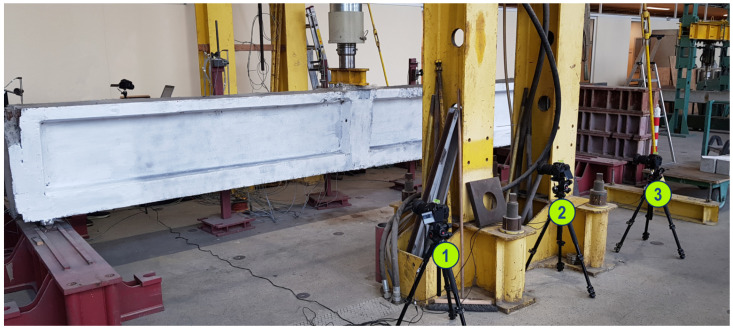
Experimental setup arranged for monitoring post-tensioned, precast crane runway beam during the three-point bending test employing vision system described in [54].

The effectiveness of the presented approach for the assessment of the precast crane beams was verified by analyzing the images taken during the laboratory evaluation of the precast, post-tensioned crane runway beams. The beams were manufactured between 1962 and 1963 and were removed from the industrial building in which they were located. The girder with an I section had a height of 80 cm and was composed of two pre-cast segments and had a total span of 600 cm. The tests were carried out on the girders at the Cracow University of Technology (CUT) Research Laboratory for Building Materials and Structures. The CivEng Vision system, developed at CUT, was used for collection, recording and analyzing the images [54,66]. Figure 20 shows the test stand prepared for the three-point bending test presenting also the numbering of acquisition devices [4].

### 5.2. Results for the Three-Point Bending Test

**Figure 21:**
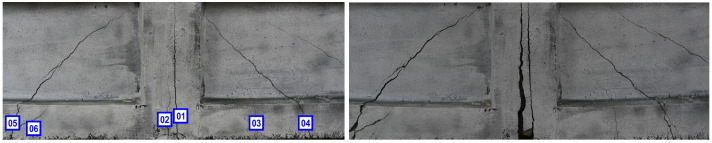
Two images of side surface of beam, captured using vision system, throughout three-point bending test, presenting also numbering of developing cracks (on the left).

Figure 21 shows two images of the lateral face of the beam taken during the course of the bending test with the vision system, presenting also the numbering of developing cracks.

**Figure 22:**
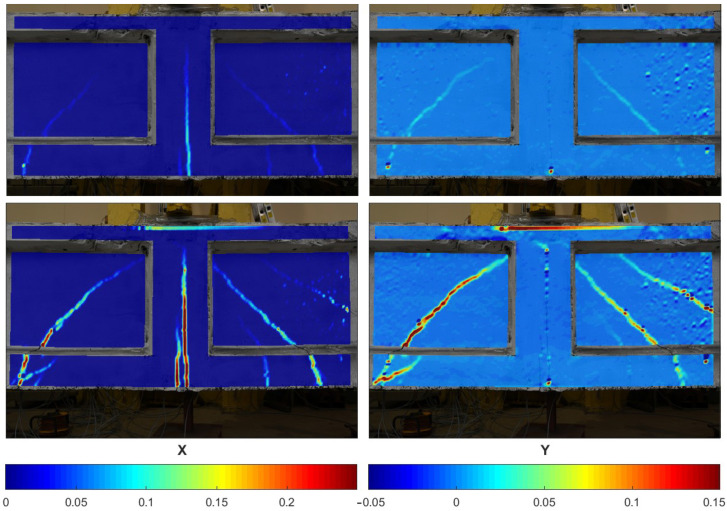
Deformation fields on beam surface in the X and Y directions during loading phase as measured by DIC, from [54].

Subsequently, the images were subjected to processing using the DIC method, and the deformation fields and width of the cracks were computed. Figure 22 illustrates the changes in the deformation fields along the X and Y axes as the beam undergoes loading.

**Figure 23:**
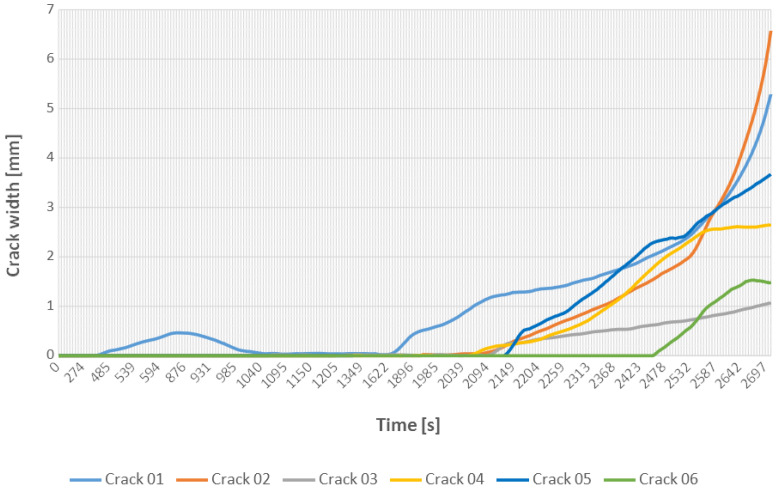
The cracks width development during the loading phase.

**Figure 24:**
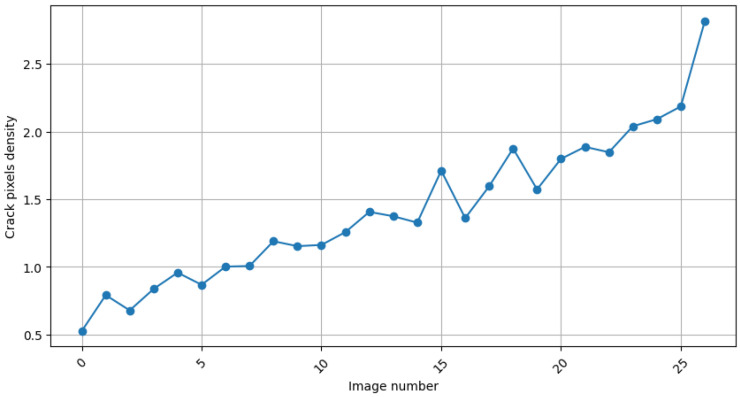
The cracks pixel density evaluation for 256 × 256 pixel frames.

As shown in Figure 23, the central vertical crack (01) developed precisely in the center of the beam. It formed at the beginning of the loading. The load then was decreased to zero and the crack was nearly fully closed. It reappeared in the course of the subsequent stage of the experiment. The images also were examined by the fine-tuned U-Net model to autonomously identify and segment the cracks present on the beam surface. Using a model fine-tuned with the SDNET dataset, image segmentation was performed. Figure 24 presents the result of the crack pixel density evaluation for 256 × 256 pixel frames.

### 5.3. Results for the Shearing Test

**Figure 25:**
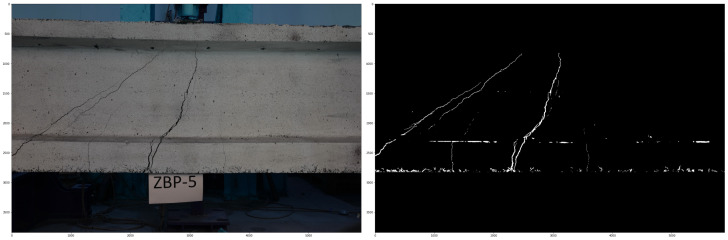
(**Left**): Image taken during laboratory experiments of the beam under shear loading, from [54]. (**Right**): Result of surface cracks segmentation after removing noise, from [62].

**Figure 26:**
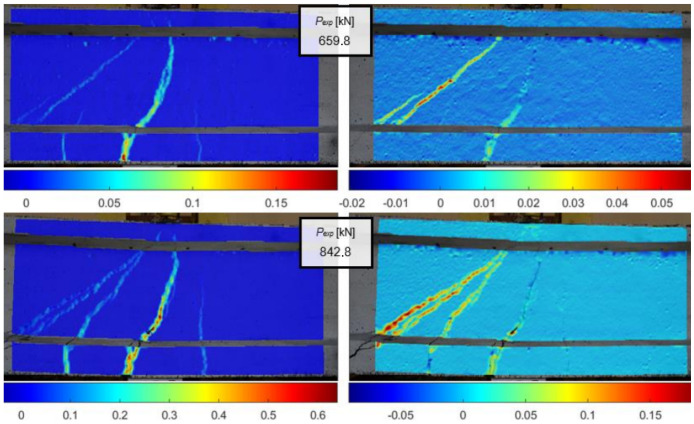
Strain full fields in X and Y axis on surface of beam under shear loading measured using DIC method, taken from [67].

The left-hand side of Figure 25 shows an image of the beam lateral surface, captured using the vision system, in the process of shear test. The images were also processed using the DIC method and the strain distributions and width of the cracks were calculated. Figure 26 presents the evolution of strain fields in X and Y axis during the load imposition of the beam.

Ultimately, the images were examined by the trained U-Net model to autonomously identify and segment the cracks on the outer layer of the beams. The right-hand side of Figure 25 presents the result of image segmentation.

While this study focuses primarily on the assessment of pre-cast concrete crane beams using advanced imaging and segmentation models, the underlying methodologies, particularly the integration of U-Net variants with digital image correlation (DIC), hold significant potential for broader applications across various structural components and materials. The methodologies can be adapted to monitor the health of bridge decks and supports, where early detection of cracks and structural weaknesses is crucial to prevent failures. In urban settings, ensuring the integrity of the facades of high-rise buildings is essential for safety. The precision of U-Net-based models in detecting tiny cracks can help in routine inspection and maintenance of these structures.

## 6. Conclusions

This study has successfully demonstrated the integration of U-Net based deep learning models with digital image correlation (DIC) for the detailed and accurate assessment of concrete precast crane beams. The research not only proves the efficacy of these technologies in detecting and analyzing cracks but also underscores their potential to improve infrastructure monitoring and maintenance practices.

This study presented a system designed for automatic evaluation of crack evolution visible on the outer layer of a concrete test sample beam under laboratory test conditions. The methodology integrates the digital image correlation (DIC) technique to track the progression of crack width with a trained U-Net convolutional neural network for automated identification and segmentation of cracks.

An analysis and comparison of U-Net model-based architectures used in image segmentation were performed, a dataset was created that can be used in tasks related to the topic, and a method for measuring cracks in concrete elements was proposed. Using the described approach allows for the automation and acceleration of the inspection process of construction objects. Knowing the exact location of the device that takes the photo, it is possible to determine the actual size of the crack.

The applied computer vision system was assessed through static tests conducted in the CUT laboratory to establish a solution capable of performing this task. The images were acquired using DSLR cameras linked to an automatic actuator. The presented system demonstrated high precision in the evaluation of surface cracks. It can automatically detect the number of cracks and determine their locations. This process is repeatable for static analyses of other concrete elements. This study aims to present a computer vision system designed to quickly assess the visible crack width on the surface of concrete test specimens, as well as to automatically detect and segment cracks for monitoring purposes.

While the presented models demonstrate high efficacy in detecting and segmenting cracks, there are several limitations. The performance of the models is highly dependent on the quality and diversity of the training data. In scenarios where crack characteristics significantly differ from those in the training set, the accuracy of the model might be degraded. The models were primarily tested under controlled laboratory conditions. Real-world applicability might see reduced effectiveness due to varying environmental factors and operational conditions that were not simulated during the training phases. Advanced architectures, especially TransUNet, require significant computational resources, which could limit their deployment in real-time applications or on portable devices that are commonly used in field inspections.

The versatility of the applied models allows for adaptation to various types of infrastructure beyond crane beams, including bridges, roads, and building facades, suggesting a wide-reaching impact in the field of civil engineering. Automating structural evaluations reduces the need for extensive manual labor and mitigates the risks associated with manual inspections, leading to significant cost savings and safety improvements. The quantitative data provided by these models supports more informed decision-making in maintenance planning. This shift towards data-driven practices helps optimize resource allocation and scheduling in maintenance operations, ultimately extending the useful life of infrastructure assets.

Looking forward, the methodologies presented here set the groundwork for further research on integrating these technologies with other non-destructive testing methods to create even more comprehensive monitoring systems. Furthermore, expanding the training data sets to include a wider range of defects and environmental conditions will improve the accuracy and generalizability of the models, making them more effective tools in the global effort to ensure infrastructure safety and resilience.

The successful application of U-Net variants and digital image correlation (DIC) in the detection and analysis of cracks in concrete precast crane beams offers promising avenues for further research and development. To expand the scope and enhance the effectiveness of this methodology, several next steps can be proposed. Integrating additional data sources such as thermal imaging, acoustic emissions, and radar scans could enrich the input of the models and provide a more comprehensive view of structural health.This comprehensive technique is capable of detecting both visible fractures and underlying flaws, as well as material deterioration, which conventional imaging techniques may miss. Extending the crack detection methodologies to incorporate 3D imaging data, such as from LiDAR or structured light systems, would allow for a more detailed analysis of crack depth and volume, which are crucial for assessing the severity of damage. Developing 3D convolutional neural network models specifically tailored for this type of data could significantly improve the accuracy and utility of crack detection in complex structural components. Using transfer learning to adapt existing models to different types of materials and environmental conditions without the need for extensive retraining. This approach can accelerate the deployment of models in new areas and reduce computational costs.

These future directions seek to broaden the scope of present methods and inspire innovation in the area of structural health monitoring. By exploring these avenues, researchers can continue to push the limits of what is possible in infrastructure maintenance, ensuring that these critical systems remain safe and functional for the communities they serve.

## Figures and Tables

**Table 1:** Summary of methods used for concrete surface crack detection.

Method	Key Features	Benefits	Drawbacks
Manual Inspection	Visual examination by experts	High detail accuracy	Subjective, time-consuming, safety risks
Linear Variable Differential Transformers (LVDT)	Mechanical measurement of displacement	Precise, reliable	Limited to visible, accessible areas
Strain Gauges	Measures deformation under load	Direct measurement of physical properties	Requires physical contact, limited coverage
Digital Image Correlation (DIC)	Optical method for full-field displacement and strain	Non-contact, high resolution	Requires high-quality imaging, complex setup
Fully Convolutional Networks (FCN)	Deep learning for semantic segmentation	Automates crack detection, scalable	Requires large labeled datasets, intensive computation
U-Net	Encoder-decoder architecture with skip connections	Precise segmentation, good with small datasets	Computationally intensive
Attention U-Net	Integrates attention mechanisms	Improves focus on relevant features	More complex than standard U-Net
TransUNet	Combines Transformers with U-Net	Captures global and local context	Very high computational resources needed

**Table 2:** Number of parameters and number of convolutional layers.

Model Name	Number of Parameters [×$10^{6}$]	Number of Conv Layers
U-Net	31.5	13
Attention U-Net	37.3	17
TransUNet	72.8	24

**Table 3:** Confusion matrix for pixel-wise crack segmentation.

	Predicted Non-Crack	Predicted Crack
Actual non-crack	TN	FP
Actual crack	FN	TP

**Table 4:** Number of parameters and training time for each model.

Model Name	# of Parameters [×$10^{6}$]	# of Trainable Parameters [×$10^{6}$]	Training Time [min]
U-Net	28.0	13.3	10
Attention U-Net	28.4	13.7	10
TransUNet	146.0	131.4	40

**Table 5:** Comparison of the performance of the three models on test data using evaluation metrics.

Model	Precision [%]	Recall [%]	F1 [%]	mIoU [%]
U-Net	86.23	86.94	86.58	90.64
Attention U-Net	85.76	86.10	85.93	90.57
TransUNet	86.18	88.08	87.12	86.03

## Data Availability

The original contributions presented in this study are included in the article. Further inquiries can be directed to the corresponding author.

## References

[1] di Marzo M., Tomassi A., Placidi L. (2024). A Methodology for Structural Damage Detection Adding Masses. Res. Nondestruct. Eval..

[2] Aalami B.O. (2023). Post-Tensioning in Building Construction.

[3] Berkowski P., Kosior-Kazberuk M. (2017). Material and structural destruction of concrete elements in the industrial environment. Procedia Eng..

[4] Derkowski W., Walczak R., Derkowski W. (2019). Problem of condition assessment of precast, post-tensioned concrete crane beams in an extended period of use. Proceedings of the Concrete—Innovations in Materials, Design and Structures: Proceedings of the *fib* Symposium 2019.

[5] Piątek B., Howiacki T., Kulpa M., Siwowski T., Sieńko R., Bednarski Ł. (2023). Strain, crack, stress and shape diagnostics of new and existing post-tensioned structures through distributed fibre optic sensors. Measurement.

[6] Brownjohn J.M. (2007). Structural health monitoring of civil infrastructure. Philos. Trans. R. Soc. Math. Phys. Eng. Sci..

[7] Spencer B.F.J., Hoskere V., Narazaki Y. (2019). Advances in computer vision-based civil infrastructure inspection and monitoring. Engineering.

[8] Frangopol D.M., Soliman M. (2019). Life-cycle of structural systems: Recent achievements and future directions. Structures and Infrastructure Systems.

[9] Koch C., Georgieva K., Kasireddy V., Akinci B., Fieguth P. (2015). A review on computer vision based defect detection and condition assessment of concrete and asphalt civil infrastructure. Adv. Eng. Inform..

[10] Mohan A., Poobal S. (2018). Crack detection using image processing: A critical review and analysis. Alex. Eng. J..

[11] Yuan Q., Shi Y., Li M. (2024). A Review of Computer Vision-Based Crack Detection Methods in Civil Infrastructure: Progress and Challenges. Remote. Sens..

[12] Garcia-Garcia A., Orts-Escolano S., Oprea S., Villena-Martinez V., Garcia-Rodriguez J. (2017). A review on deep learning techniques applied to semantic segmentation. arXiv.

[13] Munawar H.S., Hammad A.W., Haddad A., Soares C.A.P., Waller S.T. (2021). Image-based crack detection methods: A review. Infrastructures.

[14] Azouz Z., Honarvar Shakibaei Asli B., Khan M. (2023). Evolution of Crack Analysis in Structures Using Image Processing Technique: A Review. Electronics.

[15] Dorafshan S., Thomas R., Maguire M. (2018). Comparison of deep convolutional neural networks and edge detectors for image-based crack detection in concrete. Constr. Build. Mater..

[16] Islam M.M., Kim J.M. (2019). Vision-based autonomous crack detection of concrete structures using a fully convolutional encoder–decoder network. Sensors.

[17] Mousavi M., Bakhshi A. (2023). Optimized U-shape convolutional neural network with a novel training strategy for segmentation of concrete cracks. Struct. Health Monit..

[18] Fan Z., Li C., Chen Y., Wei J., Loprencipe G., Chen X., Di Mascio P. (2020). Automatic crack detection on road pavements using encoder-decoder architecture. Materials.

[19] Panella F., Lipani A., Boehm J. (2022). Semantic segmentation of cracks: Data challenges and architecture. Autom. Constr..

[20] Ai D., Jiang G., Lam S.K., He P., Li C. (2023). Computer vision framework for crack detection of civil infrastructure—A review. Eng. Appl. Artif. Intell..

[21] Dung C., Anh L. (2019). Autonomous concrete crack detection using deep fully convolutional neural network. Autom. Constr..

[22] Yang X., Li H., Yu Y., Luo X., Huang T., Yang X. (2018). Automatic pixel-level crack detection and measurement using fully convolutional network. Comput.-Aided Civ. Infrastruct. Eng..

[23] Pu R., Ren G., Li H., Jiang W., Zhang J., Qin H. (2022). Autonomous Concrete Crack Semantic Segmentation Using Deep Fully Convolutional Encoder–Decoder Network in Concrete Structures Inspection. Buildings.

[24] Shen Y., Yu Z., Li C., Zhao C., Sun Z. (2023). Automated Detection for Concrete Surface Cracks Based on Deeplabv3+ BDF. Buildings.

[25] Razveeva I., Kozhakin A., Beskopylny A.N., Stel’makh S.A., Shcherban’ E.M., Artamonov S., Pembek A., Dingrodiya H. (2023). Analysis of Geometric Characteristics of Cracks and Delamination in Aerated Concrete Products Using Convolutional Neural Networks. Buildings.

[26] Guo P., Meng X., Meng W., Bao Y. (2022). Monitoring and automatic characterization of cracks in strain-hardening cementitious composite (SHCC) through intelligent interpretation of photos. Compos. Part Eng..

[27] Xu G., Yue Q., Liu X., Chen H. (2023). Investigation on the effect of data quality and quantity of concrete cracks on the performance of deep learning-based image segmentation. Expert Syst. Appl..

[28] Forest F., Porta H., Tuia D., Fink O. (2023). From Classification to Segmentation with Explainable AI: A Study on Crack Detection and Growth Monitoring. arXiv.

[29] Ahmadi M., Lonbar A.G., Sharifi A., Beris A.T., Nouri M., Javidi A.S. (2023). Application of segment anything model for civil infrastructure defect assessment. arXiv.

[30] König J., Jenkins M., Mannion M., Barrie P., Morison G. (2022). What’s Cracking? A Review and Analysis of Deep Learning Methods for Structural Crack Segmentation, Detection and Quantification. arXiv.

[31] Feng C., Liu M.Y., Kao C.C., Lee T.Y. (2017). Deep active learning for civil infrastructure defect detection and classification. Computing in Civil Engineering.

[32] Sari Y., Prakoso P.B., Baskara A.R. (2019). Road crack detection using support vector machine (SVM) and OTSU algorithm. Proceedings of the 2019 6th International Conference on Electric Vehicular Technology (ICEVT).

[33] Zhang L., Yang F., Zhang Y.D., Zhu Y.J. (2016). Road crack detection using deep convolutional neural network. Proceedings of the 2016 IEEE International Conference on Image Processing (ICIP).

[34] Zhou S., Canchila C., Song W. (2023). Deep learning-based crack segmentation for civil infrastructure: Data types, architectures, and benchmarked performance. Autom. Constr..

[35] Deng J., Singh A., Zhou Y., Lu Y., Lee V.C.S. (2022). Review on computer vision-based crack detection and quantification methodologies for civil structures. Constr. Build. Mater..

[36] Ronneberger O., Fischer P., Brox T. (2015). U-net: Convolutional networks for biomedical image segmentation. Proceedings of the International Conference on Medical Image Computing and Computer-Assisted Intervention.

[37] Long J., Shelhamer E., Darrell T. Fully convolutional networks for semantic segmentation. Proceedings of the IEEE conference on Computer Vision and Pattern Recognition.

[38] Badrinarayanan V., Kendall A., Cipolla R. (2017). Segnet: A deep convolutional encoder-decoder architecture for image segmentation. IEEE Trans. Pattern Anal. Mach. Intell..

[39] Chen L.C., Papandreou G., Kokkinos I., Murphy K., Yuille A.L. (2017). Deeplab: Semantic image segmentation with deep convolutional nets, atrous convolution, and fully connected crfs. IEEE Trans. Pattern Anal. Mach. Intell..

[40] Rao A.S., Nguyen T., Le S.T., Palaniswami M., Ngo T. (2022). Attention recurrent residual U-Net for predicting pixel-level crack widths in concrete surfaces. Struct. Health Monit..

[41] Hang J., Wu Y., Li Y., Lai T., Zhang J., Li Y. (2023). A deep learning semantic segmentation network with attention mechanism for concrete crack detection. Struct. Health Monit..

[42] Shamsabadi E.A., Xu C., Rao A.S., Nguyen T., Ngo T., Dias-da Costa D. (2022). Vision transformer-based autonomous crack detection on asphalt and concrete surfaces. Autom. Constr..

[43] Wang W., Su C. (2022). Automatic concrete crack segmentation model based on transformer. Autom. Constr..

[44] Xiang C., Guo J., Cao R., Deng L. (2023). A crack-segmentation algorithm fusing transformers and convolutional neural networks for complex detection scenarios. Autom. Constr..

[45] Shi Z., Jin N., Chen D., Ai D. (2024). A comparison study of semantic segmentation networks for crack detection in construction materials. Constr. Build. Mater..

[46] Li H., Wang W., Wang M., Li L., Vimlund V. (2022). A review of deep learning methods for pixel-level crack detection. J. Traffic Transp. Eng. (Engl. Ed.).

[47] Słoński M. (2019). A comparison of deep convolutional neural networks for image-based detection of concrete surface cracks. Comput. Assist. Methods Eng. Sci..

[48] Sutton M., Orteu J.J., Shreier H. (2009). Image Correlation for Shape, Motion and Deformation Measurements.

[49] Pan B. (2018). Digital image correlation for surface deformation measurement: Historical developments, recent advances and future goals. Meas. Sci. Technol..

[50] Zhao J., Sang Y., Duan F. (2019). The state of the art of two-dimensional digital image correlation computational method. Eng. Rep..

[51] Peters W., Ranson W. (1982). Digital imaging techniques in experimental stress analysis. Opt. Eng..

[52] Sutton M., Wolters W., Peters W., Ranson W., McNeill S. (1983). Determination of displacements using an improved digital correlation method. Image Vis. Comput..

[53] Olufsen S.N., Andersen M.E., Fagerholt E. (2020). *μ*DIC: An open-source toolkit for digital image correlation. SoftwareX.

[54] Tekieli M. (2019). A Vision-Based Measurement System for the Analysis of Structural Element Deformation Fields. Ph.D. Thesis.

[55] Planche B., Andres E. (2019). Hands-On Computer Vision with TensorFlow 2: Leverage Deep Learning to Create Powerful Image Processing Apps with TensorFlow 2.0 and Keras.

[56] Bahdanau D., Cho K., Bengio Y. (2014). Neural machine translation by jointly learning to align and translate. arXiv.

[57] Oktay O., Schlemper J., Folgoc L.L., Lee M., Heinrich M., Misawa K., Mori K., McDonagh S., Hammerla N.Y., Kainz B. (2018). Attention U-Net: Learning where to look for the pancreas. arXiv.

[58] Vaswani A. (2017). Attention is all you need. arXiv.

[59] Chen J., Lu Y., Yu Q., Luo X., Adeli E., Wang Y., Lu L., Yuille A.L., Zhou Y. (2021). Transunet: Transformers make strong encoders for medical image segmentation. arXiv.

[60] Dorafshan S., Thomas R., Maguire M. (2018). SDNET2018: An annotated image dataset for non-contact concrete crack detection using deep convolutional neural networks. Data Brief.

[61] Team S. (2022). Segments.ai—The Training Data Platform for Computer Vision Engineers. https://segments.ai.

[62] Niemiec J. (2022). Deep Neural Networks in Structural Health Monitoring. Master’s Thesis.

[63] Sudre C.H., Li W., Vercauteren T., Ourselin S., Jorge Cardoso M. (2017). Generalised dice overlap as a deep learning loss function for highly unbalanced segmentations. Proceedings of the Deep Learning in Medical Image Analysis and Multimodal Learning for Clinical Decision Support: Third International Workshop, DLMIA 2017, and 7th International Workshop, ML-CDS 2017, Held in Conjunction with MICCAI 2017.

[64] Sha Y. (2021). Keras-Unet-Collection. https://github.com/yingkaisha/keras-unet-collection.

[65] Google (2024). Google Colaboratory. https://colab.research.google.com/.

[66] Tekieli M., Słoński M., Cecot W. (2015). Digital image correlation and Bayesian filtering in inverse analysis of structures. Recent Advances in Civil Engineering: Computational Methods.

[67] Walczak R. (2022). Shear Capacity of Concrete Crane Beams in an Extended Period of Service in Uncertain Conditions of Prestressing Cables Anchorage. Ph.D. Thesis.

